# An exploration of addiction in adults experiencing early-life stress: a metasynthesis^1^

**DOI:** 10.1590/1518-8345.2026.2939

**Published:** 2017-10-05

**Authors:** Carla Araujo Bastos Teixeira, Gerri Lasiuk, Sylvia Barton, Maria Neyrian de Fatima Fernandes, Edilaine Cristina da Silva Gherardi-Donato

**Affiliations:** 2 Doctoral student, Escola de Enfermagem de Ribeirão Preto, Universidade de São Paulo, PAHO/WHO Collaborating Centre for Nursing Research Development, Ribeirão Preto, SP, Brasil. Bolsista do Conselho Nacional de Desenvolvimento Científico e Tecnológico (CNPq), Brasil.; 3 PhD, Associate Professor, College of Nursing, University of Saskatchewan, Edmonton, Alberta, Canada.; 4 PhD, Associate Professor, Faculty of Nursing , Edmonton Clinic Health Academy, Edmonton, Alberta, Canada.; 5 Doctoral student, Escola de Enfermagem de Ribeirão Preto, Universidade de São Paulo, PAHO/WHO Collaborating Centre for Nursing Research Development, Ribeirão Preto, SP, Brasil. Assistant Professor, Universidade Federal do Maranhão, Imperatriz, MA, Brazil.; 6 PhD, Associate Professor, Escola de Enfermagem de Ribeirão Preto, Universidade de São Paulo, PAHO/WHO Collaborating Centre for Nursing Research Development, Ribeirão Preto, SP, Brazil.

**Keywords:** Child Abuse, Behavior Addictive, Adult Survivors of Child Adverse Events

## Abstract

**Objective:**

to review and synthesize qualitative research on the links between early-life stress and addiction behaviours in adulthood.

**Method:**

metasynthesis to review qualitative research findings based on procedures that outline how to identify themes or constructs across studies in a specific area. Comprehensive searches of multiple electronic databases were performed. The initial search yielded 1050 articles and the titles and abstracts were screened for inclusion based on predetermined criteria. Thirty-eight full text, peer-reviewed articles were retrieved and assessed by three independent reviewers. Twelve articles were eligible for full review and appraised using the Critical Appraisal Skills Programme (CASP) tools.

**Results:**

the findings revealed that clear associations exist between early-life stress and addictive behaviours in adulthood, such as between trauma in childhood, violence, and addictive behaviours. A common theme in the findings indicates that participants turn to addictive substances as a way of strategically coping with stressful childhood experiences, regardless of the harmful side effects or detrimental social outcomes.

**Conclusion:**

it can be inferred that addiction may be viewed as a way to deal with adversity in childhood and that there is an interrelationship between addiction, domestic violence and crime.

## Introduction

Healthy child development is built on a foundation of supportive and responsive relationships with caregivers^1^. The stress associated with the disruption or absence of these relationships when brain structures are developing, has long-term and negative effects on emotional, behavioural, social, and physical health and well-being throughout an individual’s life^2^ ^-^ ^3^.

Child maltreatment is an umbrella term for all forms of physical/emotional/sexual abuse, neglect, and exploitation that occurs before 18 years of age and is associated with actual or potential harm to the child’s health, survival, development, or dignity^4^. The following four categories of child maltreatment are typically recognized: neglect, physical abuse, psychological or emotional abuse, and sexual abuse^2^ ^,^ ^5^ ^-^ ^7^. Child maltreatment is a serious public health problem around the globe^8^ and has implications for childhood mortality and morbidity and lifelong mental health, substance misuse, high-risk sexual behaviour, obesity, and criminal behaviour^9^ ^-^ ^10^.

Rates of child maltreatment are challenging to estimate because of differences in definitions, sampling strategy, and method of data collection, as well as methodology. What is more disconcerting is that 50% - 80% of cases go unreported^8^ ^,^ ^10^. A meta-analysis of 13 independent samples (n=59,406) reported that 16.3% of children around the world are victims of physical neglect and a further 18.4% have experienced emotional neglect^11^. As many as 25% of adults report having been physically abused as children^4^. Another meta-analysis^12^ (331 independent samples and n=9,911,748 participants) estimated the international prevalence of child sexual abuse to be 12.7%. The same study found that rates of sexual abuse of females and males to be 18% and 7.6% respectively. 

 Studies suggest that the early-life associated with child maltreatment also has deleterious consequences in adulthood^13^. Adults who have experienced some form of early-life stress may present with a wide range of physical health problems, mood, anxiety, and personality disorders, and/or misuse of alcohol and other licit and illicit substances^6^ ^,^ ^14^ ^-^ ^15^. The effects of child maltreatment serve as markers of endophenotypic susceptibility to diseases through the hypothalamic-pituitary-adrenal (HPA) axis dysfunction^16^. Early-life stress can result in permanent changes in HPA axis function, morphological changes in the brain, and gene expression changes, all of which are implicated in the abuse of psychoactive substances^17^. In other words, stress in early-life may act as a catalyst for substance misuse behaviours. Stressful experiences in childhood, including physical and psychological abuses, are thought to establish a relationship with the binomial risk-resilience for the development of alcohol and drug dependence.

Although the relationship between early-life stress and the misuse of psychoactive substances has been substantiated in the basic science literature, an appreciation of the psychosocial processes involved goes beyond statistical causality. The meaning of life events to individuals and the links to subsequent behaviours are difficult to illustrate through quantitative research studies. A metasynthesis of qualitative research on addiction in adults who have experienced early-life stress will provide additional knowledge and a deeper understanding of the psychosocial and intrapersonal dynamics of addictive behaviours. It addresses the following question: What does the qualitative research literature reveal about the experience of early childhood stress and the presence of addictive behaviour in adulthood? 

## Aim

The purpose of this metasynthesis was to explore studies on early-life stress and links to addiction in adulthood. The specific procedural steps were to search, appraise, classify, and synthesize the findings of qualitative research in order to describe the existence of addiction among adults with links to the experience of early-life stress.

## Method

### Design

Metasynthesis is an approach to reviewing qualitative research findings based on procedures that outline how to identify themes or constructs across studies in a specific area. This metasynthesis involved a comprehensive search, appraisal of the results of qualitative studies, study classification, and synthesis of the results^18^. These procedures were chosen because it allows the researcher to build a set of relevant assumptions, aggregating the results of several primary studies; and to discover the “state of the art” whereby the contributions of combining qualitative research findings enhances their contribution to the development of new knowledge and future knowledge applications^19^ ^-^ ^22^. The goal of a metasynthesis is to broaden and deepen the understanding of a particular phenomenon^23^.

### Search method

Comprehensive searches of Medline, PsycINFO, CINAHL, Web of Science and SCOPUS databases were performed. Primary search terms included subject headings and text words associated with key concepts related to the review question. The terms were divided into three broad categories: 1) *child abuse* (“trauma” “Battered Child Syndrome”, “adverse experience”, “aggression”, “forced sex”, “child abuse”, “victim”, early life experience”); 2) *addiction/misuse of substances* (“substance-related disorders”, “addiction”, “substance misuse”, “overconsumption”); and 3) *qualitative studies* (“qualitative research”, ”anthropology”, “ethnography”, “hermeneutic”, “phenomenology”, “lived experience”, “grounded theory”). The search terms were refined during the search process and various combinations of subject headings and keywords were used depending on the database and the controlled vocabularies available; no limits were placed on publication dates. The searches were conducted in July and August 2015. The peer-reviewed articles were retrieved and assessed by three independent reviewers. In case of disagreement in any of the phases, the articles were re-read and discussed until the disagreement was resolved. EndNote^®^ was used to organize and manage references. Inclusion/exclusion criteria were developed using a modified PICOS framework^24^: Population: Adults (18 years and older) who self- -report early-life stress. Phenomenon of Interest: Meaning/subjective description of the experience of addiction. Context: Individuals who report having early-life stress and who have experienced addiction in adulthood. Outcome: Subjective descriptions of addiction. Study design: Metasynthesis of findings of primary qualitative research studies.

### Quality Appraisal

Quality appraisal of studies in the review was performed using Critical Appraisal Skills Programme (CASP) tools. Recommended by the Joanna Briggs Institute, the CASP Qualitative Research Checklist tool offers a standardized approach for evaluating the rigour of qualitative studies^25^. The Checklist consists of 10 questions: two for the selection of studies and eight for the design of research, data collection and analysis, ethics, reflexivity and implications of qualitative research^26^. According to the authors, the first three questions are fundamental. If for any of them the answer is “no,” the article has to be excluded; thus the article is considered outside the required methodological standards criteria and is left out.

### Data Extraction

The instrument elected to extract the data was adapted^27^ and has been used in earlier studies^28^ ^-^ ^29^. The adapted items used were author, title, keywords, journal, database, country, year, aim, methods, findings, reference and additional information. The reviewers independently reviewed the extracted data using this instrument, which consists of five domains: identification of the study, setting of study, journal, methodological characteristics of the study, and quality appraisal.

### Data analysis

Employing a method^30^ that the data were analyzed in the following way: (i) initial reduction/classification of the data into systematic categories; (ii) clustering of primary source data through an interactive contrasting, comparing process; and (iii) drawing conclusions about each sub-group analysis and synthesizing relevant elements into an integrated summary^31^.

### Validity

The validity for this meta-synthesis was grounded in a comprehensive search for literature, group discussion on search terms and inclusion criteria^18^, group assessment of appraisal of CASP, and agreement about decisions. The authors also discussed findings of the studies and themes until a consensus was reached.

## Results

**Figure 1:**
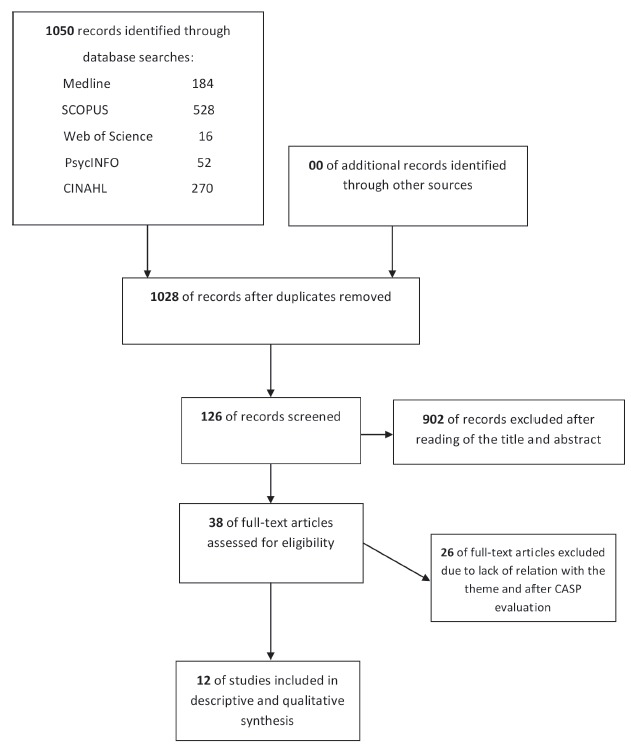
Flow chart of study selection for meta-synthesis.

**Figure 2:**
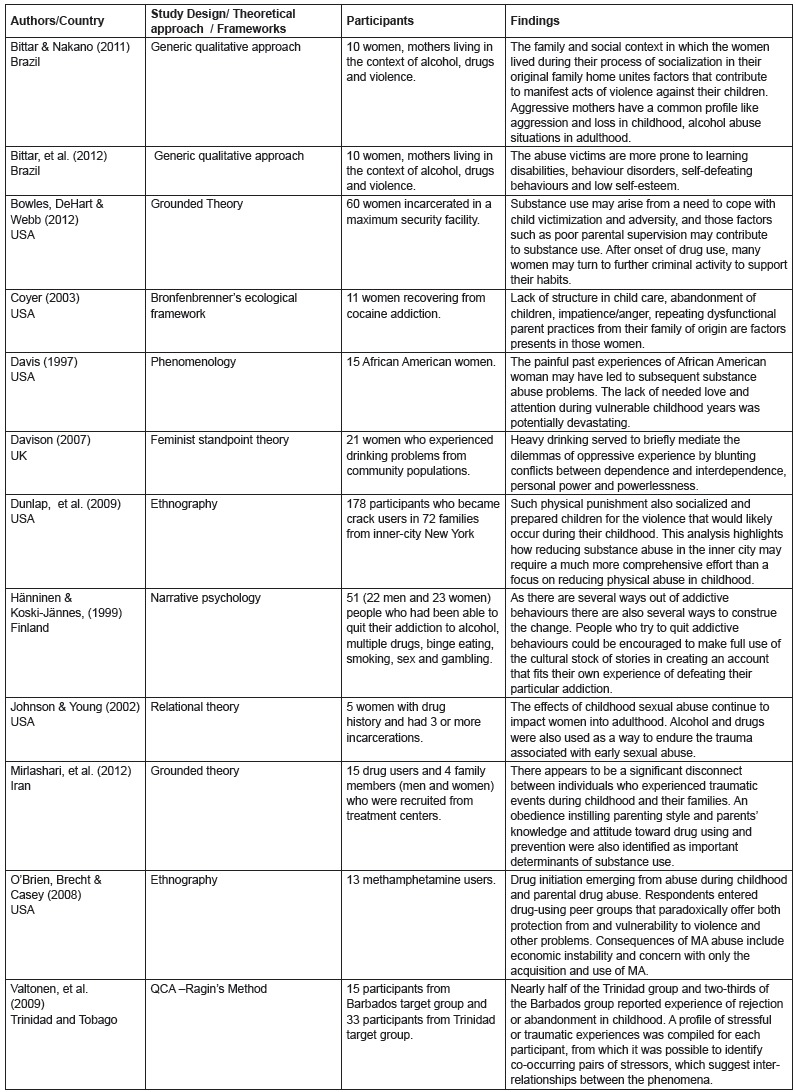
Studies included in the meta-synthesis

The initial search generated 1050 records; 22 duplicates were removed. Preliminary screening of titles resulted in the elimination of an additional 929 records. The abstracts of the remaining 126 records were screened and 88 of these were discarded. The full texts of 38 records were reviewed and the 12 articles that met all inclusion criteria were retained for full review (See Figures 1 and 2).

### Sources of Early-Life Stress

Participants in the studies in this review reported a variety of sources of early-life stress and no single subtype stood out. Participants described early-life stress associated with physical, emotional/psychological, and sexual abuse; physical neglect; and emotional neglect. Others reported stress related to parental loss, divorce, and abandonment. In all studies, early life stress was self-reported. There was no use of instruments to measure child abuse.

The findings of this metasynthesis support the claim that for many people, adult addiction is closely related to early-life stress. The major theme across all 12 studies in this review is that use of psychoactive substances, gambling, and sex served as ways of coping with stressful circumstances experienced at an early age. In some studies^32^ ^-^ ^36^ participants report engaging in addictive behaviours in childhood which continued into adulthood. Regardless of when the addiction behaviour began, the common factor among participants is that addiction in adulthood is a way of coping with the effects of early-life stress. 

In eight of the twelve studies^32^ ^-^ ^34^ ^,^ ^37^ ^-^ ^41^, alcohol was identified as the drug of abuse. Five studies^32^ ^-^ ^35^ ^,^ ^40^ addressed poly-substance use; two^35^ ^-^ ^36^ addressed the use of methamphetamine; two^33^ ^,^ ^42^ focused on crack use; three articles^32^ ^,^ ^41^ ^,^ ^43^ focused on cocaine use; and one study^35^ addressed opium and heroin use. An article^40^ focused on process addiction, including sex addiction, smoking, binge eating, alcohol misuse and gambling, and the misuse of chemical substances. The others highlighted adverse situations experienced in childhood as an important factor in the misuse of chemical substances. 

A notable finding is the existence of relationships between addiction, crime, and domestic violence. Nine of the twelve studies^32^ ^-^ ^33^ ^,^ ^35^ ^-^ ^39^ ^,^ ^41^ ^,^ ^43^ identify domestic violence as a fertile setting for child abuse and substance abuse. Two studies^32^ ^,^ ^34^ describe crime as the final point in life trajectories marked by childhood maltreatment and substance misuse. Two studies^32^ ^,^ ^34^ focused specifically on the experiences of incarcerated persons and the abuse of substances.

## Discussion

This metasynthesis provides insight into the experience of addictive behaviour in adulthood in relation to the occurrence of early life stress. The studies included in this review reveal that addiction in adult life is a way of coping with the trauma of stressful experiences in childhood. Another important finding was the relationship between trauma in childhood, violence, and addictive behaviours. 

### Addiction as a Coping Strategy

Coping, defined as a set of cognitive-behavioural actions developed by the individual throughout their life course experiences, develops as a result of many stressors, in order to change adverse aspects in the environment and regulate potential threats arising from these^44^. Coping strategies are used to deal with demands or stressors (internal or external) that a person judges as being above their resources^44^. It is understood, therefore, that the means an individual uses to cope may change over time, according to the features of the stressor and contextual factors^45^.

Faced with a stressful situation, individuals develop different ways of coping, which are related to personal factors, situational demands and available resources; and aim to restore the balance of the organism to the reactions triggered by the stressor. The types of coping strategies used in specific situations are in accordance with an individual’s personality, past experience, and the characteristics of the situation^46^ ^-^ ^47^.

According to the Transactional Model of Stress, the person-environment relationship is a dynamic interaction between a person and his or her environment and stress results from a perceived imbalance between an individual’s resources and the demands placed on them. The Model also assumes that the individual brings to every event or situation beliefs, values, attitudes, and behaviours developed over a lifetime under the influence of genetic, personal, social, and environmental factors. These contribute to a worldview and shape the meaning, value, and significance ascribed to a particular situation or event and helps to explain why two individuals may perceive and react differently to the same circumstances^48^.

The coping processes constitute a mobilization of cognitive and behavioral efforts to manage (reduce or tolerate) the internal or external demands that arise from the interaction with the environment^48^. These coping efforts may be either problem-focused or emotion-focused. The way a person perceives a stressful situation (i.e., cognitive appraisal) influences how they will attempt to cope^49^ ^-^ ^50^.

Problem-focused coping focuses on changing the environment to eliminate or modify the stressful situation. In other words, the person seeks to understand the stressor and tries to modify it. Emotion-focused coping is aimed at alleviating the emotional distress experienced by the person. That is, the person tries to mitigate the suffering related to the stimulus^51^. Emotion-focused coping can be considered a way of avoiding direct confrontation with the stressor and referred to as avoidant coping.

Studies included in this review focused on childhood experiences, which would be considered as traumatic. This finding is aligned with the results of other studies, including a study^52^ with families and children about the use of substances that connect physical, emotional and sexual abuse to a sense of degradation, humiliation and drug use. Also, individuals who suffered multiple maltreatments had low self-esteem and were involved in more risk behaviours, including increased use of alcohol and other drugs^53^.

In an attempt to avoid the negative feelings, participants used the addiction behaviours as a way of trying to escape painful memories or limit stressful situations, what is referred to as avoidant coping. There’s a relationship between early life stress, educational level, and the use of avoidant coping in the misuse of substances and mental disorders in women^54^.

 Avoidant (emotion focused) coping does not solve problems caused by stressors. It is a way around the problem, which can cause further consequences such as addiction, crime and violence, for example. Addiction is just one of the many problems faced by these individuals, and the interplay of addiction, negative relationships, and violence can contribute to the misuse of substances as a means of escape from adverse circumstances.

Considering the linkage among addiction, crime and domestic violence, a person who developed addiction behaviour as a coping strategy can create a cycle of early-life stress across the generations. Two major themes were apparent in terms of avoidance coping: 1) repeating dysfunctional patterns from the family of origin, and 2) pain caused by traumatic memories.

### Repeating Dysfunctional Patterns from the Family of Origin 

Most often, children in the reviewed studies accepted the abuse from their relatives and did not try to retaliate, even if they were physically capable of doing so. This was way out of the realm of experiencing love and respect. In fact, these children accepted that being abused was a part of being a child. In justifying their punishments, they came to define parental assault as culturally normative as revealed in some of the reviewed articles^37^ ^-^ ^38^ ^,^ ^41^ ^,^ ^43^ ^,^ ^55^.

Often, there is no self-reflection about this behaviour, which leads to a repetition of the dysfunctional pattern of abuse and addiction within the family environment. As the person has no knowledge of how to cope otherwise with the traumatic events he/she is experiencing, they continue to adopt and repeat the same behaviours learned within the family environment.

It was recognized that some participants were using the same learned behaviour as a method of discipline that their parents had used. Unfortunately, it is noted that their development and ability to process family interactions are likely to be impaired with predictable outcomes; and that such situations have potential for perpetuating the patterns of vulnerability and risk for these adults. This creates a vicious cycle of abuse and addictive behaviour.

### Forgetting Pain Caused by Traumatic Memories

The addiction behaviours are an understandable attempt to decrease the emotional and mental pain caused by such traumatic and violent experiences. The individuals disclosed experiences of abuse, incest, rape and many other horrors that lead to drug and alcohol use, in order to extinguish pain as revealed in many of the reviewed^32^ ^-^ ^36^ ^,^ ^39^ ^-^ ^40^ ^,^ ^55^.

The participants in the reviewed studies used an array of different drugs to find balance in a life of pain. A lack of self-esteem, the tendency toward depression, and the feelings of shame and inadequacy set the stage for addiction behaviour. Indeed, it was a form of self-medication, because with these substances can come some temporary relief to the pain of recalling severe and traumatic memories.

As the traumatic past memories cause pain, the use of substances can be seen as a way to alleviate the suffering. The substance abuse is viewed as a means to feel more comfortable at facing, emotionally, the deleterious consequences of child abuse. It is a non-assertive way of coping with the situation in the same way that the analgesic does not cure, only brings temporary but immediate relief. This does not solve the trauma, quite the opposite, as the misuse of substances can bring forth more complex problems that result from addiction.

However, the addiction behaviour is often perceived as a unique recourse for handling the trauma. In other words, this is an opportunity for health professionals, especially those who engage in community work, to become aware of childhood trauma indications and to offer new resources to this at risk population.

### Addiction, Violence, and Crime

One of the major findings from the present study is that violence was commonplace in the household. Prior examination of literature reveals that household violence is part of a complex of sub-cultural norms that can involve drug use and sexual exploitation. Such a scenario can be seen in other qualitative studies, revealing the problem of violence in the context of misuse of substances^55^ ^-^ ^56^.

The result of substance abuse together with the social context in which people find themselves, as well as the absence of family or support systems, may set up a dynamic between parent and child which can increase risks of early-life stress. This cycle of addiction-violence-addiction is perpetuated, passing from parents to children, being renewed, constantly. The number of children affected by parental substance misuse, globally, is a social issue requiring action at many organizational levels. 

In addition to domestic violence, addiction may be seen as a catalyst for crime, or a way to sustain an addiction behaviour or addictive life that has been influenced by traumatic events experienced since childhood within the family environment. Different scenarios are seen, whereby people who grew up in the middle of the violence use this to maintain the addiction. Sometimes the violence is a result of addiction, and viewed as a complication. Other times, the drug trade keeps the circle of violence, crime and addiction behaviour going. As such, family structure and parenting dynamics emerged as important themes by which to understand these pathways to addiction in adulthood. We can see, within this review, which individuals were from disruptive homes. Their stories indicated that childhood experiences can be traumatic and may continue to affect their life course, including the presence of addictive behaviours.

The reason that addiction leads to crime is varied, sometimes family addiction can lead to difficulty paying bills as parents use all their resources to support their habits, and resort to crime along the way. Addiction led to a lot of difficulty holding down jobs, and with unemployment came the start up of criminal activity to support drug habits.

In other words, it is known that, sometimes, criminal activity, early-life stress and addiction behaviour are closely related. Thus, preventive actions targeted at the identification of child abuse, can prevent not only the harmful consequences of addiction, but also prevent lower levels of violence related to this phenomenon. In other words, if it is possible to act preventively in child abuse cases, it is possible to prevent addiction behaviours, domestic violence and criminal activities. 

In providing assistance to individuals exhibiting addiction behaviour, it is crucial to consider the presence of early stressors as a feature of great importance in the life history of the person. Identifying and accepting such stressful experiences in childhood can help the health professional and client better understand the meaning of these behaviours; thus enabling a redefinition or reframing of trauma experiences and a search for coping strategies that are more constructive in nature.

### Relevance for clinical practice 

The growth of children encompasses physical, emotional, cognitive and psychological existence. Parents can support this growth in many ways, sometimes positively, other times not. They can contribute positively to the child’s sense of security, ability to form healthy relationships, and ability to become a productive adult^57^. When there is a lack of this support, the child needs quality care and sustained attention in order to avoid deleterious consequences in the future. 

Nurses working in community and/or pediatric areas are in an ideal position to identify children affected by child maltreatment and to assist them in finding the help they need, including addiction prevention or treatment if the child or teen is already exhibiting addiction behaviour to cope with their adversities. Consequently, nurses are able to make a difference in the approach to challenges like early-life stress and addiction behaviour through a comprehensive or holistic nursing lens that considers the social, environmental, personal, and health dimensions of each person’s situation. 

In addition, nurses can provide advanced mental health care in terms of family needs related to physical and psychological health, healing, and wellbeing based on an assessment process and action plan derived from knowledge of the client’s life that includes stories of early-life stress and addiction. 

Community resources must be made available to those individuals having experienced adverse events such as child abuse. Nurses working throughout the healthcare system have the capacity to connect those in need and their families with appropriate resources, as well as help them navigate a system that leads to obtaining the care they require. Lastly, this study opens a window for further research that seeks a deeper understanding of the connections between early life adverse experiences, human addictive behaviour, and trauma-informed care that could be harnessed through the professional discipline of nursing, as well as through interdisciplinary work with the health sciences. 

## Limitations

One of the limitations of this study is the lack of access to transcripts of the included material. Due to it being a metasynthesis, access to the transcripts of the participants could bring greater depth to the discussion, which limits the interpretation of synthesized information. In spite of these limitations, the metasynthesis revealed some promising results that can inform and guide effective health promotion and disease prevention actions in nursing practice.

## Conclusions

The metasynthesis review provides an understanding of the link between the experience of addiction and early-life stress. It can be inferred that addiction may be viewed as a way to deal with adversity in childhood and that there is an interrelationship between addiction, domestic violence and crime. The results are useful to clinicians, nurses and health professionals seeking a deeper understanding of individual perspectives related to adverse life events. This knowledge can be incorporated into practice, leading further to new links between research and treatment.

This metasynthesis highlights several areas for future research. There is a clear indication that increasing the focus on understanding addiction behaviours in persons who have experienced early-life stress, in the context of their perceptions and understandings, would be valuable. At the same time, it is imperative that concerted efforts be made to improve the quality of life of this population through health promotion, disease prevention, and intervention strategies specific to mental health.
